# Sterigmatocystin Occurrence in Paddy and Processed Rice Produced in Italy in the Years 2014–2015 and Distribution in Milled Rice Fractions

**DOI:** 10.3390/toxins9030086

**Published:** 2017-02-28

**Authors:** Terenzio Bertuzzi, Marco Romani, Silvia Rastelli, Annalisa Mulazzi, Amedeo Pietri

**Affiliations:** 1 Food & Feed Science and Nutrition Institute, Faculty of Agriculture, Università Cattolica del Sacro Cuore, Via Emilia Parmense, 84 – 29122 Piacenza, Italy; silvia.rastelli@unicatt.it (S.R.); annalisa.mulazzi@unicatt.it (A.M.); amedeo.pietri@unicatt.it (A.P.); 2 Ente Nazionale Risi Rice Research Centre, Strada per Ceretto, 4 –Castello d’Agogna, 27030 Pavia, Italy; m.romani@enterisi.it

**Keywords:** sterigmatocystin, paddy rice, processed rice

## Abstract

The occurrence of sterigmatocystin (STC) in paddy and processed rice samples produced in Italy was surveyed. After extraction and purification, STC was analysed using HPLC-MS/MS. STC was detected in all paddy rice samples (*n* = 49), in the range 0.29–15.85 μg·kg^−1^. As regards processed rice, a widespread contamination was found in brown and parboiled rice. All the brown rice samples were contaminated between 0.12 and 1.32 μg·kg^−1^; for parboiled rice, the incidence was 90.9% and the maximum level was 1.09 μg·kg^−1^. The contamination in white rice was significantly lower (*p* < 0.01). The STC distribution in different rice fractions, obtained by the de-hulling and polishing processes, was evaluated. After de-hulling, the STC percentage remaining in brown rice was in the range 21.2%–30.8%. The polishing process, from brown to white rice, caused another remarkable decrease of contamination; the STC remaining in white rice was 2.2%–8.3% of the amount found in paddy rice.

## 1. Introduction

**Figure 1:**
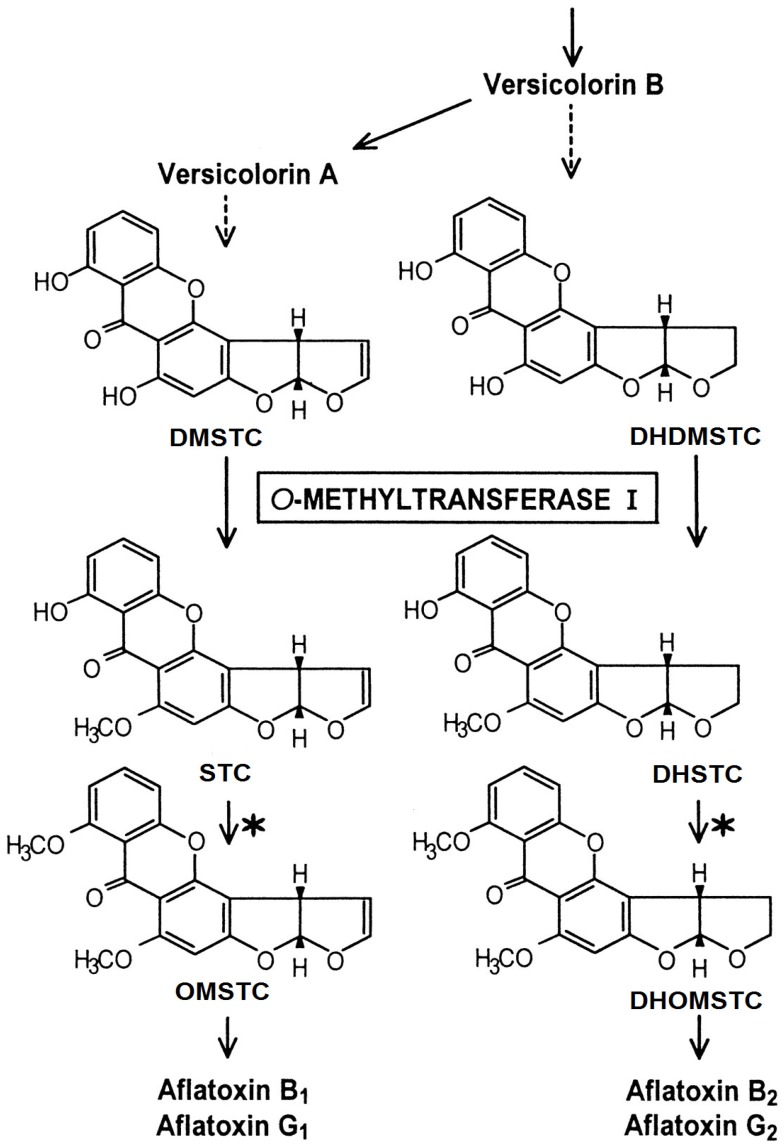
Biosynthesis of sterigmatocystin (STC) and, depending on the fungal species, further to aflatoxins.

Sterigmatocystin (STC) is a mycotoxin produced by fungi of the genus *Aspergillus* (mainly *A. nidulans* and *A. versicolor*) as well as by other species belonging to the genera *Bipolaris*, *Chaetomium* and *Emiricella*; *A. versicolor* is the most common source in food. STC shares its biosynthetic pathway with aflatoxins (Figure 1); *A. nidulans* and *A. versicolor* seem unable to metabolise STC into O-methylsterigmatocystin, the direct precursor of aflatoxin B_1_ and G_1_. As a consequence, food commodities infested by these fungi can contain high amounts of STC; on the contrary, infestation by *A. flavus* and *A. parasiticus* can cause low amounts of STC, because most is converted into aflatoxins [1,2,3]. STC has an aflatoxin-like structure including a furofuran ring system; in several studies STC was recognised as a potential carcinogen, mutagen and teratogen in animals [4,5,6,7]. In 1987, STC was categorised by the International Agency for Research on Cancer (IARC) as a class 2B, possible human carcinogen [8]. Recent studies showed that STC forms DNA adducts after metabolic activation to an epoxide at the furofuran ring [9] and is more genotoxic than AFB_1_ in three types of human cell lines [10]. To date, European legislation has no fixed limits for STC occurrence in food; only the Czech Republic and Slovakia have set limits of STC at 5 μg·kg^−1^ for rice, vegetables, potatoes, flour, poultry, meat, milk and 20 μg·kg^−1^ for other foods. No health-based guidance value (HBGV) has been established for STC.

STC can occur in grains and grain-based products [11,12] and in other food such as green coffee, nuts, spices, beer and on the surface of ripened cheese [13,14,15,16,17]. STC is generally analysed by chromatographic techniques (HPLC-UV, HPLC-FLD, LC-MS/MS, GC-MS). In the most recent surveys, the preference was given to LC-MS/MS methods for the determination of STC in different food matrices, such as cereals, beer, cheese, nuts and feed [13,18,19,20,21]; STC was also detected by multi-mycotoxin LC-MS/MS methods [22,23,24]. Some LC-MS/MS methods proposed direct analysis of crude extracts, whilst others developed different clean-up steps, such as SPE or immunoaffinity column [20,21,25].

A call for data on STC launched by EFSA in 2010 resulted in only a limited number of results for food samples (247), which were mostly lower than LOD. Successively, the results of a survey on STC in food highlighted that STC was rarely present in cereal grains and cereal products (limit of quantification of 0.5 μg·kg^−1^), except paddy rice and derived products [26]; in fact, all paddy rice samples (*n* = 28), mainly originating from Italy and Greece, were contaminated with STC. Contamination of rice with STC has already been reported [27,28,29,30]; however, the number of analysed samples was low and high limits of quantification were often reported. 

Italy is the principal rice producing country in Europe; in 2014, the area devoted to rice growing (mainly located in northern Italy) covered about 227,000 ha and rice production amounted to nearly 1,473,000 tons [31]. Given the relevance of Italian production and the high incidence of STC in rice, this study was aimed at providing more data on STC contamination in paddy, brown, parboiled and white rice produced in Italy over two years (2014–2015) and evaluating STC distribution in the fractions obtained during the rice de-hulling and polishing process. 

## 2. Results and Discussion

### 2.1. Occurrence of STC in Paddy and Processed Rice

Descriptive statistics (incidence, mean, median and maximum value) of the results are reported in Table 1. For the samples falling between LOD and LOQ, we tentatively calculated the value by proportion with the lowest calibration standard. All the results were corrected for the average recovery values.

As regards paddy rice samples, STC was detected in all samples in the range of 0.29–15.85 μg·kg^−1^. No significant difference between STC levels in samples harvested in 2014 and 2015 was found (*p* = 0.571). The percentage of samples showing a value over 1.0 μg·kg^−1^ was similar in 2014 and 2015 (55.6% and 54.8%, respectively); considering both years, the percentage of samples exceeding the value of 1.0 and 2.0 μg·kg^−1^ was 55.1% (*n* = 27) and 32.6% (*n* = 16), respectively. Only 2 samples (one for each year) showed a contamination value higher than 5.0 μg·kg^−1^, the EU limit for AFB_1_ in maize and rice to be subjected to sorting or other physical treatment before human consumption [32].

In a recent European survey on STC occurrence in cereals, rice was clearly identified as the product with the highest incidence of contamination [26]; we collaborated in that study and found that all paddy rice samples grown in Italy during 2013 (*n* = 13) were contaminated (similar results were obtained for paddy rice from other countries). In that limited survey, the average contamination and the median were 0.89 and 0.76 μg·kg^−1^, respectively; the STC level was higher than 1.0 μg·kg^−1^ in 38.5% of the samples and the maximum value was 1.9 μg·kg^−1^. The analytical method used in that study was similar to that described in this work (only the clean-up was modified, as reported in Section 4.5, then a comparison of the data obtained from both surveys is possible; no significant difference between the three years, 2013–2015, was found (*p* = 0.273). These results showed that contamination with STC in paddy rice grown in Italy is not occasional, but widespread each year. The samples of this study were collected at harvest time (generally in October), then it is reasonable to hypothesize that the contamination occurred during growth in the fields. 

The paddy rice samples exceeding the STC value of 3.0 μg·kg^−1^ (*n* = 9) were analysed for AFs determination, according to the method of Bertuzzi et al. [33]. AFB_1_ was detected in only 1 sample at a low level (0.2 μg·kg^−1^; LOD = 0.05 μg·kg^−1^), showing that STC contamination is probably independent of AFs contamination, even if they share the biosynthetic pathway. 

As regards processed rice samples, contamination levels in brown, parboiled and white rice were significantly lower than in paddy rice (*p* < 0.01). For brown rice, all the samples were contaminated between 0.12 (value < LOQ) and 1.32 μg·kg^−1^; in 12.5% of the samples (*n* = 3), the STC level was higher than 1 μg·kg^−1^. For parboiled rice, the incidence was high (90.9%), but only one sample (4.5%) exceeded 1 μg·kg^−1^; no significant difference was found between brown and parboiled rice collected in both years (*p* = 0.399). As regards white rice, the contamination level was low: 24.3% of the samples (*n* = 9) were uncontaminated and STC values between the LOD and LOQ were found in 45.9% (*n* = 17) of them. Finally, STC never exceeded 1 μg·kg^−1^. Contamination in white rice was significantly lower than in brown and parboiled rice (*p* < 0.01). In the recent European survey [26], 89 processed rice samples were analysed. STC was detected in 21% of samples (*n* = 19), in particular, in 50% (5 of 10) and 14% (11 of 76) of brown and white rice samples, respectively; the mycotoxin exceeded 1 μg·kg^−1^ in 50% of the contaminated brown rice samples, whilst the maximum value in white rice was 0.68 μg·kg^−1^. In that study, the incidence of STC in processed rice samples was remarkably lower if compared with that obtained in this survey (21% vs. 76%). This difference is mainly due to the lower LOD value (LOD: 0.05 vs. 0.10 μg·kg^−1^); in fact, 12 samples of white rice collected in this study (32.4%) showed a contamination in the range of 0.05–0.10 μg·kg^−1^.

STC occurrence in processed rice was reported in few other surveys, often in a limited number of samples. Remarkable incidence and high levels of STC were reported by Rofiat et al. [30] in processed rice from Nigeria; STC was detected in 17 of 38 samples (44.7%), with a mean value of 19 μg·kg^−1^ (median of 0.75 μg·kg^−1^) and a maximum value of 125 μg·kg^−1^. On the other hand, no STC contamination was found in 48 brown rice samples collected in Japan [29] and in a wide range of rice collected at retail outlets in the UK [34]. The recent review of Sempere Ferre (2016) on mycotoxin occurrence in rice [35] showed that very few studies reported data on contamination with STC [36,37].

### 2.2. Distribution of STC during De-Hulling and Polishing Processes

**Figure 2:**
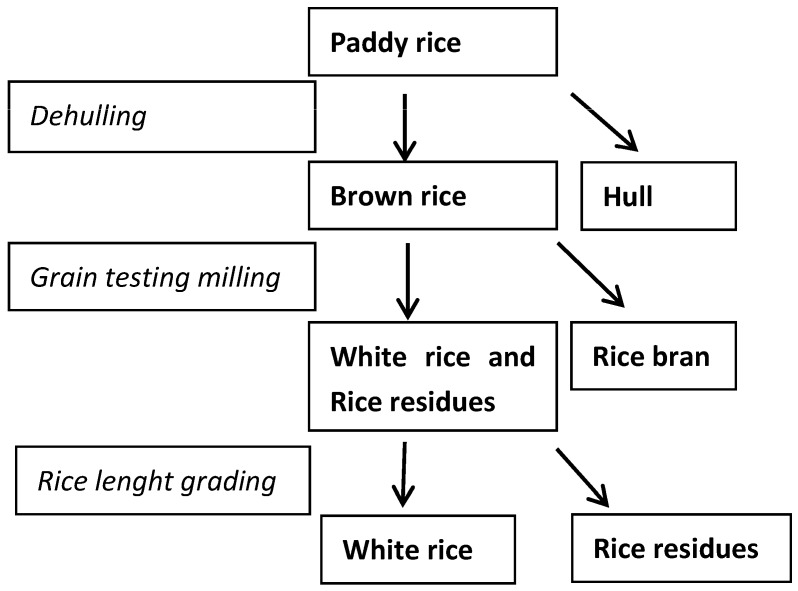
Layout of the polishing processes.

STC concentration in different rice fractions, obtained by the de-hulling and polishing processes (*n* = 5, Figure 2), is reported in Table 2.

The mass balance of the STC amount during the overall process [(Σ STC fractions/Σ STC paddy rice) × 100] was calculated; it was satisfactory, in the range 83.1%–98.1% (average 88.5% ± 5.9%). The de-hulling process resulted in a remarkable decrease of contamination; only a percentage between 21.2% and 30.8% of STC initially occurring in paddy rice remained in brown rice. The highest amount was in the hull, fractions used neither in food nor in feed because of the high silica content. Considering the concentration values, the concentration factor paddy->brown rice, calculated as (STC conc. in brown rice)/(STC conc. in paddy rice), varied from 0.26 to 0.38; on average, the concentration in brown rice was a third of that in paddy rice (Table 3). The polishing of brown rice causes a remarkable decrease of STC in white rice. This STC decrease may be in the range of 62.9%–92.7%. Regarding paddy rice, only 2.2%–8.3% of the STC present remained in white rice. The ratio (STC conc. in white rice)/(STC conc. in brown rice) ranged from 0.10 to 0.49, with a mean of 0.24 ± 0.15.

## 3. Conclusions

This study indicates widespread occurrence of STC in paddy rice produced in Italy; the levels are generally low, but, considering the toxicity of the mycotoxin, further research about its incidence and the optimal conditions for its production should be carried out. Co-occurrence of STC with AFB_1_ seems very unlikely. The data obtained from the survey and from the laboratory-scale treatments, show that rice processing causes a strong reduction of STC, mainly for white rice. Particular attention to brown and parboiled rice contamination should be paid. Brown rice is sometimes used as an ingredient for breakfast cereals and snacks, often for consumption by children; it contains healthy compounds that are removed in white rice and its use brings benefits to the consumer; however, a high quality of this product should be ensured. The parboiling process improves the nutritional profile and texture of rice; however, only the bran layer is removed and therefore the STC contamination is analogous to that of brown rice. In conclusion, STC occurrence in Italian rice products should be constantly monitored; moreover, further surveys on rice produced in other countries could help to evaluate the spread of STC contamination.

## 4. Materials and Methods

### 4.1. Sampling

A total of 132 samples, including 49 paddies and 83 processed rice (24 brown, 22 parboiled and 37 white rice) were collected from October 2014 to April 2016; all the samples originated from crops grown in 2014 and 2015 in Northern Italy, in an area that accounts for more than 90% of total Italian rice production. Sampling was carried out according to the guidelines for the official control of foodstuffs as described in Commission Regulation (EC) No. 401/2006 [38]. Paddy rice samples were collected from farms and storage facilities; an aggregate sample of 5-8 kg for each field was collected and dehydrated to below 8% moisture. Processed rice samples were collected from wholesale and retail sources. A total of 4 units were collected; depending on the size of the unit (0.5 or 1 kg), the aggregate sample varied from 2 to 4 kg.

### 4.2. Rice Processing

Five lab-scale polishing processes on paddy rice were undertaken (Figure 2) as follows: paddy rice (about 0.5 kg) was de-hulled (de-huller G390/R, Colombini & Co. Srl, Abbiategrasso, Milano, Italy) by passing through a rubber-roll huller consisting of a couple of Teflon-covered rolls, and separated into brown rice and hull. Then, an aliquot (about 300 g) of brown rice was polished using a grain testing mill (TM-05, Satake Engineering Co., Tokyo, Japan), obtaining rice bran, white rice and rice residues (broken kernels). Finally, white rice and broken residues were separated through passage into a rice length grader (TRG, Satake Engineering Co., Tokyo, Japan); different graders were used, depending on the rice varieties. 

All the samples were milled using a cyclone hammer mill (1 mm sieve, Pulverisette, Fritsch GmbH, Idar-Oberstein, Germany) and homogenised. After milling and homogenisation, an aliquot (50 g for rice-milling fractions; 200 g for rice) of the sample was taken and stored at −20 °C until the time of analysis.

### 4.3. Chemicals and Standards

Chemicals and solvents used for the extraction and clean-up were ‘pro-analysis’ quality or better; solvents and reagents for instrumental analysis were LC-MS/MS grade. The composition of the phosphate buffer (PBS) was as follows: NaCl 8 g·L^−1^, KCl 0.2 g·L^−1^, Na_2_HPO_4_ 1.15 g·L^−1^, KH_2_PO_4_ 0.2 g·L^−1^ and pH 7.4. The STC analytical reference standard was obtained from Sigma-Aldrich (St. Louis, MO, USA; purity 98.5%); the internal standard [^13^C_18_]-sterigmatocystin (96.4% ^13^C) was purchased from Biopure (Tulln, Austria) as the standard solution in acetonitrile (1.2 mL, 25.7 μg·mL^−1^, uncertainty 1.02 μg·mL^−1^). Stock STC standard solution was prepared in ethanol at a concentration of 10 mg·L^−1^; the solution was calibrated spectrophotometrically at 325 nm using the value 16,218 L·mol^−1^·cm^−1^ for the absorption coefficient [39] and stored at −20 °C when not in use; working standard solutions were prepared by dilution with acetonitrile-water (40/60 *v*/*v*). Five STC standards, mixed with isotopically labelled STC standard solution (12 μg·L^−1^; 90/10 *v*/*v*), in the range between 0.06 and 1.0 μg·L^−1^ were injected.

### 4.4. Analysis for STC Determination

STC was extracted from an aliquot of 20 g taken from the milled sample with 100 mL acetonitrile-water 80/20 *v*/*v* using a rotary-shaking stirrer for 60 min. After filtration through a folded filter paper, 2 mL of filtrate was diluted with 20 mL of PBS and cleaned using an immunoaffinity column (R-Biopharm-Rhône, Glasgow, UK); this clean-up step was already tested by Marley et al. [25] for STC determination in beer. After washing of the column with 2 mL water, STC was eluted in a graduated glass vial with 6 mL acetonitrile. The extract was concentrated under a gentle flow of nitrogen and brought to 1 mL with acetonitrile-water 40/60 *v*/*v*. This volume (1 mL), corresponded to 0.4 g of the sample. An aliquot of 900 μL of cleaned extract was transferred into an autosampler vial and mixed with 100 μL of isotopically labelled STC (12 μg·L^−1^). A volume of 20 μL of the extract was injected into an LC-MS/MS system consisting of a LC 1.4 Surveyor pump, a Quantum Discovery Max triple-quadrupole mass spectrometer (Thermo-Fisher Scientific, San Jose, CA, USA) and a PAL 1.3.1 sampling system (CTC Analytics AG, Zwingen, Switzerland). The system was controlled by Xcalibur 1.4 software (Thermo-Fisher). The instrumental analysis was carried out as reported by Mol et al. [26]. After separation on a Betasil RP-18 column (5 μm particle size, 150 × 2.1 mm, Thermo-Fisher) with a gradient acetonitrile-water (both acidified with 0.2% formic acid; flow rate 0.2 mL·min^−1^), the ionisation was performed using positive atmospheric pressure chemical ionisation (APCI) as follows: voltage 4.0 kV, sheath and auxiliary gas 29 and 5 psi, respectively, temperature of the heated capillary 270 °C. The mass spectrometric analysis was performed in selected reaction monitoring (SRM). For fragmentation of the [M + H]^+^ ions (*m*/*z* 325 and 343 for STC and [^13^C_18_]-STC, respectively), argon was used as collision gas at the pressure of 1.5 mTorr. For STC, three transitions were measured: *m*/*z* 310 (24 V) [quantifier], 281 and 253 (35 V) [qualifiers]. For the isotopic label, the transitions were: *m*/*z* 327 (24 V) [quantifier], 297 and 268 (35 V) [qualifiers]. As regards quantitative determination, linear calibration (equal weighting, ignore origin) was performed. Since the internal standard was added after extraction and clean up, it corrected for the matrix effect only, not for recovery.

### 4.5. Method Performances

The chosen method for STC quantification was developed and published in the study of Mol et al. [26]; in that study, validation parameters and inter-laboratory reproducibility were evaluated. In this study, we only replaced the use of an OASIS column with an immunoaffinity column in the clean-up step; however, some performances of the method were again evaluated.

#### 4.5.1. Matrix Effect

The matrix effect may occur due to the presence of compounds in extracts that can co-elute with the analyte, affecting the ionisation of the analyte. It can be compensated most effectively by the use of isotopically labelled STC as an internal standard; by normalising the response of STC for its labelled internal standard, it is possible to use solvent standards for calibration of the different products analysed. Then, the internal standard calibration was applied, adding the isotope-labelled -[^13^C_18_]-STC standard to all sample extracts and calibration standards. The matrix effect, calculated by comparing the response of isotopically labelled STC in sample extracts and in the standard solution, was not very pronounced (less than 10%).

#### 4.5.2. Calibration and Quantification 

**Figure 3:**
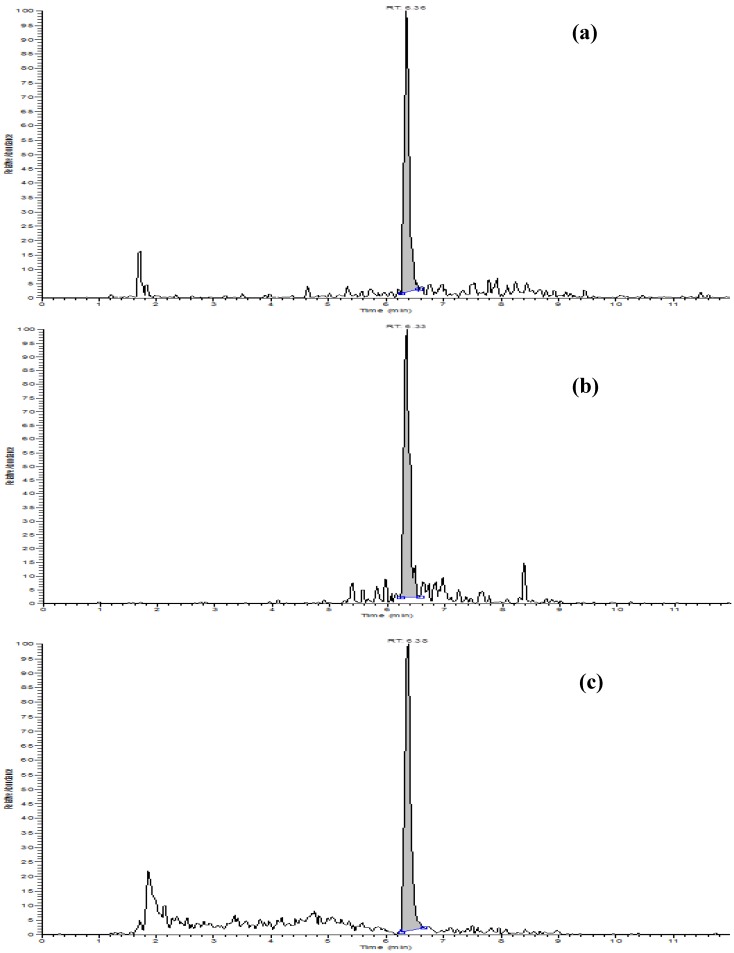
Chromatograms (transition *m*/*z* 325–310) of: (**a**) sterigmatocystin (STC) calibration standard in solvent (0.06 μg/L, corresponding to the LOQ, 0.15 μg/kg); (**b**) naturally contaminated sample at 0.16 μg/kg; (**c**) naturally contaminated brown rice sample at 0.54 μg/kg.

The linearity of the LC-MS/MS measurement was established through five calibration standards in solvent to which the labelled internal standard was added at a fixed concentration (1.2 μg·L^−1^). The concentration levels of the calibration standards were: 0.05, 0.10, 0.4, 1.0 and 2.0 μg·L^−1^; for hull and rice bran, a standard at 4.0 μg·L^−1^ was added. Linearity of calibration curves was always satisfactory, as proved by the coefficient of determination values (r), always above 0.996. The limit of detection (LOD) and the limit of quantification (LOQ) were determined by the signal-to-noise approach, defined as those levels resulting in signal-to-noise ratios of 3 and 10, respectively. The analytic response and the chromatographic noise were both measured from the ion chromatograms of blank sample extracts fortified with an appropriate volume of STC standard solution. The LOD and the LOQ were low: 0.05 and 0.15 μg·kg^−1^, respectively. The clean-up of the sample extract through immunoaffinity column removed most of the compounds that can co-elute with the analyte, reducing the matrix effect and enabling attainment of a very low instrumental noise (Figure 3).

#### 4.5.3. Accuracy and Precision 

The accuracy of the method was established by determination of the recovery. Recovery experiments were performed on paddy rice and white rice by spiking uncontaminated (blank) samples, previously milled and homogenised, with an appropriate volume of STC standard solution at three different levels (0.5, 1.0 and 2.0 μg·kg^−1^); three replicates were analysed for each level. For hull and rice bran, two levels were considered, 5.0 and 10.0 μg·kg^−1^. Each matrix was also analysed without spiking, as well as a reagent blank. The spiked sample was allowed to stand for two hours at ambient temperature under a fume hood to allow any residual solvent to evaporate.

Satisfactory recovery values were obtained, fulfilling the performance criteria fixed by EC Regulation 657/2002 (recovery between 70%–110% for analyte contamination levels from 1 to 10 μg·kg^−1^) [40]. The average recoveries were 90.4% ± 4.2%, 90.9% ± 5.1%, 92.8% ± 3.6%, 87.3% ± 3.8% and 88.1% ± 2.5% for paddy rice, brown rice, white rice, hull and rice bran, respectively.

### 4.6. Statistical Data Analysis 

Statistical analysis of mycotoxin contamination data was carried out after common logarithm transformation. This is suggested when the variance of data is higher than the mean. Statistical analysis was run using the IBM SPSS statistics package (ver. 23, 2015 Inc., Chicago, IL, USA). One way ANOVA was applied to evaluate significant differences; data were compared using the post-hoc Tukey Test. 

## Figures and Tables

**Table 1:** Incidence, mean, median and range (μg·kg^−1^) of sterigmatocystin in paddy rice, brown, parboiled and white rice produced in Italy in the years 2014–2015.

Sample	Year	Incidence	Mean	Median	Range
**Paddy rice**	2014	18/18	1.65	1.15	0.29–5.32
	2015	31/31	2.06	1.11	0.36–15.85
	Overall		1.91 ^a^	1.15	
**Brown rice**	2014	14/14	0.48	0.29	0.12–1.32
	2015	10/10	0.46	0.34	0.13–1.10
	Overall		0.47 ^b^	0.32	
**Parboiled rice**	2014	9/10	0.46	0.39	<0.05–1.09
	2015	11/12	0.30	0.27	<0.05–0.66
	Overall		0.37 ^b^	0.31	
**White rice**	2014	18/27	0.12	0.06	<0.05–0.98
	2015	10/10	0.20	0.21	0.09–0.30
	Overall		0.14 ^c^	0.08	

^a, b, c^ Values marked by different letters within a column are significantly different (*p* < 0.01).

**Table 2:** Results of five lab-scale polishing processes on four rice varieties; yield of rice fractions, sterigmatocystin (STC) concentration and relative distribution in rice-milling fractions referred to paddy rice and (between parentheses) to brown rice.

Sample	Loto ^a^ (Long-Grain A) ^b^			CL 26 ^a^ (Long-Grain B ) ^b^			Sole ^a^ (Short-Grain) ^b^			Selenio ^a^ (Short-Grain) ^b^			Sole ^a^ (Short-Grain) ^b^		
	% (*w*/*w*)	STC μg·kg^−1^	Distrib. (%)	% (*w*/*w*)	STC μg·kg^−1^	Distrib. (%)	% (*w*/*w*)	STC μg·kg^−1^	Distrib. (%)	% (*w*/*w*)	STC μg·kg^−1^	Distrib. (%)	% (*w*/*w*)	STC μg·kg^−1^	Distrib. (%)
**Paddy rice**	100.0	3.47	100	100.0	1.15	100	100.0	1.27	100	100.0	0.46	100	100.0	1.56	100
**Hull**	18.2	10.89	57.1	19.4	4.03	67.8	18.3	4.91	70.8	17.3	1.58	58.7	18.3	6.56	76.9
**Brown rice**	81.8	1.32	30.8 (100)	80.6	0.31	21.7 (100)	81.7	0.33	21.2 (100)	82.7	0.17	30.4 (100)	81.7	0.43	22.4 (100)
**Rice bran**	9.3	8.46	22.5 (72.9)	9.7	1.84	15.6 (72.0)	9.4	1.98	15.0 (70.4)	9.5	0.92	18.9 (62.1)	9.4	2.00	12.2 (54.3)
**White rice**	60.5	0.13	2.2 (7.3)	61.6	0.06	3.2 (14.8)	61.1	0.07	3.4 (15.9)	65.0	<LOD	-	61.1	0.21	8.3 (37.1)
**Rice residues**	12.1	0.32	1.1 (3.6)	9.3	0.08	0.6 (2.8)	11.2	0.13	1.2 (5.5)	8.2	0.11	1.9 (6.4)	11.2	0.13	1.0 (4.3)

^a^: rice variety; ^b^: EU classification.

**Table 3:** Average relative distribution (mean ± std) and average concentration ratio (conc. in fraction/conc. in paddy rice) of sterigmatocystin (STC) in rice-milling fractions referred to paddy rice.

Sample	STC Distribution (%)	Concentration Ratio
Paddy rice	100	1
Hull	66.2 ± 8.3	3.62 ± 0.42
Brown rice	25.1 ± 4.8	0.31 ± 0.06
Rice bran	16.8 ± 4.1	1.77 ± 0.45
White rice	4.4 ± 2.3	0.07 ± 0.04
Rice residues	1.1 ± 0.4	0.11 ± 0.06
